# Regulatory framework for development and marketing authorization of allergen products for diagnosis of rare type I and type IV allergies: The current status 

**DOI:** 10.5414/ALX02505E

**Published:** 2024-05-31

**Authors:** Julia Zimmer, Vera Mahler

**Affiliations:** Paul-Ehrlich-Institut, Division of Allergology, Langen, Germany

**Keywords:** allergology, type I allergy, type IV allergy, diagnosis, regulation, quality, safety and efficacy, skin prick test, patch test

## Abstract

Development, production, and marketing authorization of allergen products is generally challenging due to several specific characteristics, including the natural source as well as the multitude of allergenic materials. Also, depending on the frequency of sensitization in the population, the number of patients available for inclusion in clinical trials can be a limiting factor for product development. In the development of allergen products for diagnosis of type I and type IV allergies these challenges are particularly demanding because, in contrast to certain products for allergen-specific immunotherapy, no exemptions from marketing authorization are foreseen for this product group in Directive 2001/83/EC. Thus, the regulatory framework is constantly adapted within the legal scope in order to balance necessary regulatory requirements ensuring quality, safety, and efficacy with the clinical need for a comprehensive range of diagnostic allergen products. In this article, we give an overview on the current regulatory framework for development and marketing authorization of allergen products for diagnosis of rare type I and type IV allergies.

## Introduction 

Four types of hypersensitivities known as type I to type IV are distinguished according to Coombs and Gell [23, 32]. While type I, II, and III are antibody-dependent, type IV hypersensitivities are characterized by a cell-mediated response. Allergic contact dermatitis is a frequent disease manifestation of delayed-type IV hypersensitivity [3, 25], in which exposure to environmental chemicals (haptens) and their subsequent binding to endogenous proteins (haptenization) ultimately results in type IV allergen-specific sensitization via a complex interplay of immune cells [26]. Upon re-exposure and antigen presentation, T cells secret cytokines, thereby initiating an inflammatory cascade causing a delayed-type skin reaction (i.e., erythema, infiltration, papules, vesicles) usually limited to the site of contact with the respective allergenic substance. In type I allergy in contrast, after sensitization to a specific proteinaceous allergen, an immediate allergic reaction (e.g., rhinitis, rhinoconjunctivitis, urticaria, bronchial asthma, and/or anaphylactic reactions) manifests upon re-exposure to a specific proteinaceous type I allergen via cross-linking of immunoglobulin (Ig) E antibodies on sensitized mast cells and subsequent mediator release [22]. 

The range of allergen sources and allergens with the ability to cause type I allergy is enormous and includes, e.g., plant pollens, foods, insect venoms, drugs, molds, and animal products [4]. An even wider range of allergens has been associated with type IV allergy [24]. Consequently, meaningful diagnosis in patients affected by either type I or type IV allergy necessitates a comprehensive range of diagnostic allergen products (i.e., in vivo test allergens). 

## Use and availability of test allergens for diagnosis of type I and type IV allergies 

The gold standard in type IV allergy diagnosis is epicutaneous patch testing [29]. Patch test preparations contain type IV allergens (haptens) in a suitable matrix for in vivo application on the patients’ skin. In contrast, for diagnosis of type I allergy besides in vivo test allergens (e.g., skin prick test (SPTs), intradermal test and provocation test preparations) containing allergen extracts of natural origin, in vitro blood tests for quantification of allergen-specific IgE are available (either containing natural extracts of allergen sources, purified natural or recombinant allergens) [27]. While in vivo test allergens are medicinal products (drugs) [15], in vitro test allergens are medical devices and are regulated accordingly [14]. 

**Figure 1:**
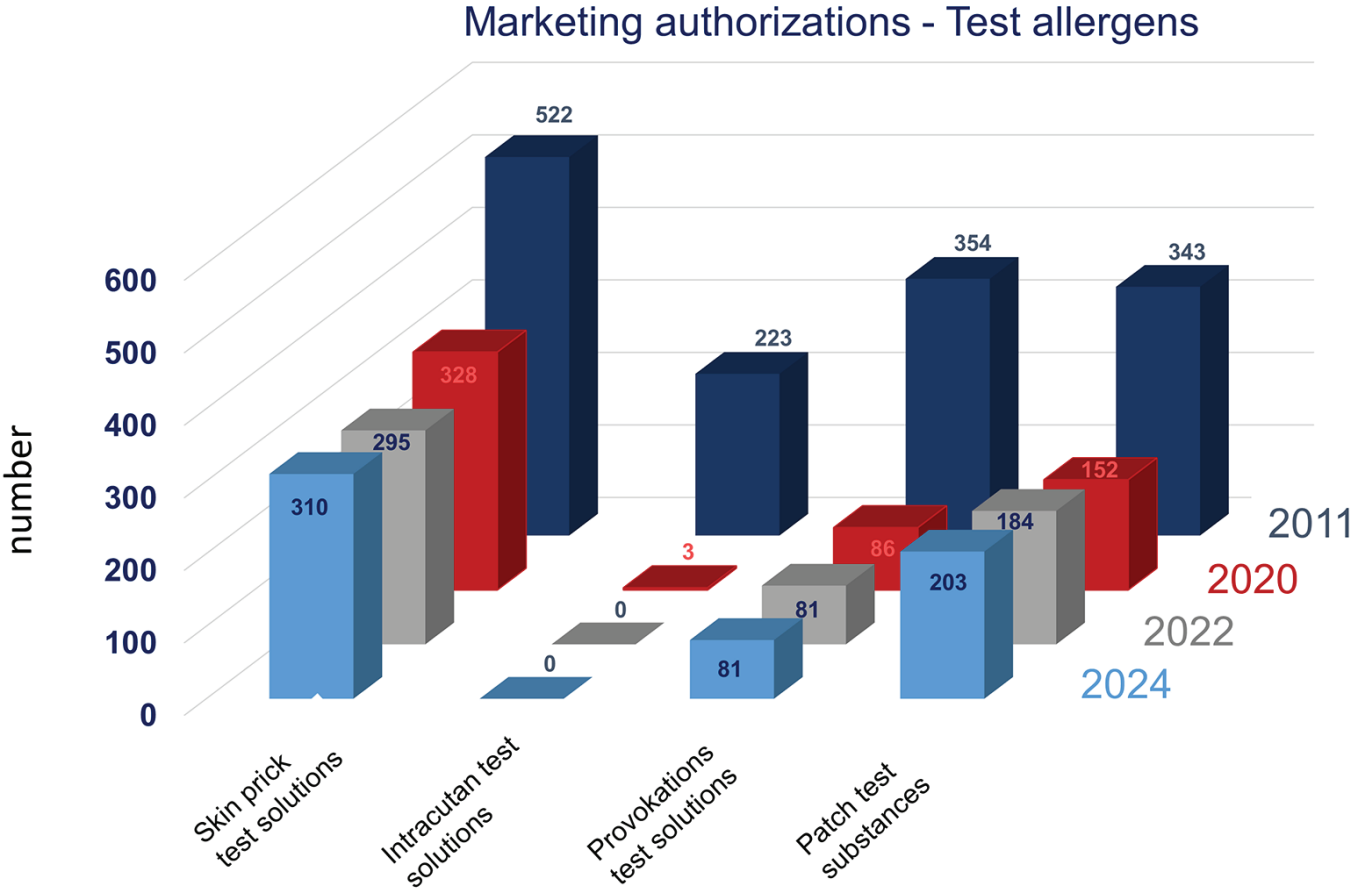
Test allergens with marketing authorizations in Germany (as of March 19, 2024).

For type I and type IV allergies alike, the number of diagnostic allergen products available to the physicians has significantly decreased in many EU member states over many years, especially affecting products for diagnosis of less common allergies [28]. In Germany, a massive loss of approved test allergens took place between 2011 and 2020, which has fortunately not continued with the same speed in the last years and has been countered by approval of dozens of epicutaneous patch test preparations and several prick test solutions (Figure 1). Nevertheless, the situation is tense because many authorized test allergen products are currently not marketed by the marketing authorization holder. Especially in the field of rare allergen sources it appears doubtful whether or not the allergen product manufacturers will expand their reduced product portfolios again. Main reasons commonly communicated for the reduction of available diagnostic allergen products are an imbalance between production costs and revenue as well as regulatory requirements, especially for products from less common allergen sources [31]. 

The aim of this paper is to outline the current regulatory framework for allergen products for diagnosis of type I as well as type IV allergies, and to give an overview how regulators have consistently adapted regulatory requirements within the legal framework to accommodate for the special characteristics of test allergen products. Although the majority of regulatory information provided is applicable EU-wide, the authors´ background is a German perspective. It is also important to note that the focus of this paper is solely on allergen products manufactured in an industrial production process, whereas allergen products produced, e.g., under the responsibility of a physician are not discussed. Likewise, specifics of innovative medicinal allergen products manufactured using, e.g., recombinant DNA technology or synthetic peptides are not covered. 

## Regulatory framework – legal basis and guidelines 

First and foremost, it is important to emphasize that Article 1 of Directive 2001/83/EC (as amended) unambiguously defines that all products used for in vivo diagnosis of allergies are medicinal products [15]. Consequently, according to Article 2 of the same Directive, a marketing authorization (MA) is required before such products may be placed on the market, if they are either prepared industrially or manufactured by a method involving an industrial process. In contrast to therapeutic allergen products which, depending on the respective national legislation, may be marketed as named patient products (NPPs) according to Article 5 of the Directive if prescribed for individual patients under the direct responsibility of a physician, there exist no such exemptions for diagnostic allergen products. This is due to the fact that diagnostic allergen products (dispensed in multi-dose containers) are generally neither manufactured or formulated in response to an order of an authorized healthcare professional, nor is their use limited to an individual patient. 

Although these fundamental regulatory requirements have been implemented more than 20 years ago, due to different application of Article 5 and individual use of exemptions, the current authorization status of allergen products remains heterogeneous among members states. Based on these exemptions, there are still allergen products of unknown quality and efficacy in the European market. However, several activities have been launched in the last years to harmonize the regulatory situation in the EU with regard to allergen products [1, 2, 7]. As a result, more and more member states have taken on national efforts to improve and harmonize the regulatory situation. Thus, step by step, the number of allergen products of unknown quality and efficacy on the market is reduced. 

In order to prove sufficient quality and efficacy, general requirements for the data to be submitted for marketing authorization application (MAA) are laid down in the Directive 2001/83/EC, in Article 8 (3). However, it should be noted that apart from a full stand-alone MAA, different kinds of applications are possible, depending on the feasibility to obtain clinical data for a specific product [1, 2]. For example, for certain allergen products for in vivo diagnosis where only a very limited number of patients exist, so-called *mixed MAA applications* are possible based on a mixture of clinical/non-clinical study reports and bibliographic data in line with Annex I, Part II, Section 7 of Directive 2001/83/EC. Furthermore, for epicutaneous patch test preparations, the *well-established use MAA* (based on bibliographic clinical data, only) according to Article 10a of the Directive has been widely used as alternative legal basis already, but in certain cases this might also be an option for type I test allergens [8]. 

The respective data submitted for MAA should generally be in line with current guidance documents applicable to the different types of allergen products, of which the most important ones have been summarized in Table 1 for diagnostic products for type I and type IV allergy. The main regulatory requirements laid down in these guidance documents are outlined below differentiating between test allergens for type I and type IV allergies; due to their substantial differences in nature, pathomechanism of induced allergic reactions, medicinal use, and risks, different aspects apply in regulatory procedures. 

## Quality, non-clinical and clinical requirements for allergen products for in vivo diagnosis of type I allergy 

### Quality 

The European Pharmacopoeia (Ph. Eur.) represents the cornerstone of quality requirements of any medicinal product in Europe. Depending on the characteristics and mode of application of a product, different monographs apply. For example, microbiological quality of allergen products for in vivo diagnosis of type I allergy like SPTs have to be tested according to the Ph. Eur. *Method of Analysis 2.6.1 Sterility*. As these products are based on aqueous extracts prepared from the respective source material, they also fall in the scope of the *Monograph on Allergen Products* [19]. The central aim of the monograph is to ensure identity, content, and reproducible quality of allergen products, with the so-called in-house reference preparation (IHRP) as centerpiece. The IHRP is a representative preparation used to verify batch-to-batch consistency, which originally has undergone biological standardization and needs to be characterized as extensively as possible, including detection and quantification of relevant allergens wherever possible. Overall, the Ph. Eur. requirements for diagnostic allergen products are generally similar to the requirements for therapeutic allergen products, but a potency test is not strictly mandatory for diagnostic products. In cases of a low prevalence of a particular allergy and thus limited feasibility to establish certain elaborate analytical methods, Ph. Eur. conform batch control of diagnostic allergen products for type I allergy can mainly comprise of a comparison of the protein profile with the respective IHRP to ensure the identity and control of protein content (80 – 120% of the stated amount) as late as possible in the manufacturing process. However, sound justification has to be provided for all additional test methods listed in the monograph that are not applied to a specific product. In addition to the *Monograph on Allergen Products* applicable to all extract-based allergen products, monographs for the most common allergenic source materials have been established in 2017, covering pollens, Hymenoptera venoms, mites, animal epithelia and outgrowths as well as molds [16, 17, 18, 20, 21]. Allergen products for in vivo diagnosis of type I allergy have to comply with these monographs if they are based on the corresponding source materials. 

The *Guideline on Allergen Products: Production and Quality Issues* mirrors the requirements of the Ph. Eur. *Monograph on Allergen Products* but complements its regulatory requirements with more detailed information [9]. Both documents have been specifically tailored to accommodate for the specific challenges associated with allergen products due to their biological origin. One of the most important aspects of the guideline is the introduction of the concept of homologous groups. Although it has to be noted that compared to preclinical and clinical aspects the degree of flexibility regarding quality requirements is somewhat limited, because these are mostly independent of allergy prevalence, a certain degree of extrapolation is possible regarding stability data and process validation. Usually, the *Guideline on Process Validation for Finished Products* requests that process validation data needs to be generated for each medicinal product to prove the suitability of the corresponding manufacturing process [13]. However, in view of the vast number of allergenic source materials, the respective regulatory requirements have been adapted to enable extrapolation based on the concept of homologous groups, thus reducing the number of process validations. This facilitation is further explored by the *Guideline on Allergen Product Development for Immunotherapy and Allergy Diagnosis in Moderate to Low-Sized Study Populations*, which is currently in public consultation [8]. Meeting the needs of a field with such a multitude of source materials, the current draft of this guideline describes the possibility of extrapolation beyond homologous groups, provided certain prerequisites are fulfilled and respective justification is provided. 

### Non-clinical/clinical aspects 

As stated above, the Directive 2001/83/EC defines the extend of data to be submitted for MAA. The main requirements for toxicological and pharmacological data are laid down in Part 3 of Annex I, generally describing the necessity of tests regarding toxicity, mutagenic and carcinogenic potential as well as analysis of pharmacodynamics and pharmacokinetics. However, products intended for diagnosis of type I allergy are based on native extracts prepared from natural allergenic source materials like pollen or mites, which are prevalent in nature and can generally be regarded as harmless for non-allergic individuals. Thus, as described in the current draft *Guideline on Allergen Product Development for Immunotherapy and Allergy Diagnosis in Moderate to Low-Sized Study Populations*, non-clinical data can usually be limited to relevant bibliographic data in conjunction with a profound expert statement, discussing the general risks of product application and a subsequent benefit risk assessment [8]. 

Part 4 of Annex I of Directive 2001/83/EC covers the requirements for clinical documentation of medicinal products to ensure their clinical safety and efficacy, but the central guideline for clinical development of diagnostic allergen products for type I allergy is the *Guideline on Clinical Evaluation of Diagnostic Agents* [11]. This guideline provides detailed information on the development of diagnostic agents as well as the design of the corresponding clinical trials to ensure that adequate technical and diagnostic performance in relation to a standard of truth^1^ or comparator can be demonstrated. However, in development of diagnostic allergen products, the available patient population might limit the applicability of this guideline. The current draft *Guideline on Allergen Product Development for Immunotherapy and Allergy Diagnosis in Moderate to Low-Sized Study Populations* outlines in which cases clinical development can deviate from the *Guideline on Clinical Evaluation of Diagnostic Agents* [8]. This includes alternatives for the normally mandatory determination of sensitivity and specificity under special circumstances as well as the suitability of biological standardization trials according to the Nordic Guidelines [30] or Turkeltaub [34] to serve as dose-finding study. In general, it has to be noted though that despite the options outlined in the new guideline draft, evidence should generally be provided at the highest possible level of confidence. Furthermore, compared to quality aspects, the concept of homologous groups introduced in the *Guideline on Allergen Products: Production and Quality Issues* has more far-reaching consequences for the clinical development [9]. 

[^1^“Standard of truth is believed to give the true state of a patient or the true value of a measurement. It provides an independent way of assessing the same variable being assessed by the investigational diagnostic agent. (…)” [11]]. 

## Quality, non-clinical and clinical requirements for allergen products for in vivo diagnosis of type IV allergy 

### Quality 

Compared to allergen products for diagnosis of type I allergy, epicutaneous patch test preparations for diagnosis of type IV allergy are not based on extracts of naturally occurring allergenic source materials. The active substances are usually chemicals which are mixed directly into either petrolatum or water. Thus, these products are neither in the scope of the *Monograph on Allergen Products* nor the *Guideline on Quality of Allergen Products*. Nevertheless, general regulatory requirements for medicinal products apply (Table 1) and specifications controlling the finished product have to verify identity, homogeneity, and active substance content. Given the commonly very broad product portfolios of epicutaneous patch test manufacturers, it is especially important that unambiguous identification is ensured compared to, e.g., chemically similar products in the portfolio by selection of suitable analytical methods. The wide range of active substances utilized in epicutaneous patch test preparations also affects other regulatory requirements. For example, in some cases the products contain active substances for which a Ph. Eur. monograph exists (e.g. ampicillin). In these cases, the active substance has to be acquired in Ph. Eur. quality. However, many other starting materials used for the production of epicutaneous patch test preparations are “atypical active substances” derived from chemical industries outside of the pharmaceutical legislation, like dyes, adhesives, metals, or fragrances. Quality requirements have been adapted accordingly, accepting that Good Manufacturing Practice (GMP) requirements are commonly not fulfilled by these substances. Instead, GMP requirements only apply after reception of the material at the medicinal product manufacturer. 

Also, the great variety of epicutaneous patch test preparations has been taken into account by regulators with regard to process validation, which may deviate from the *Guideline on Process Validation for Finished Products* [13]. The draft of the *Guideline on Allergen Product Development for Immunotherapy and Allergy Diagnosis in Moderate to Low-Sized Study Populations* describes the corresponding matrix approach that may be applied [8]. Provided an identical manufacturing process is used, the guideline describes the possibility to group different epicutaneous patch test preparations for process validation according to central product characteristics like dosage form, batch size, and concentration range, thus limiting process validation to a limited number of representative products. 

### Non-clinical/clinical aspects 

As already outlined, Part 3 of Annex I of Directive 2001/83/EC defines the extent of data to be submitted for MAA regarding toxicology and pharmacology. In case of epicutaneous patch test preparations, chemical substances commonly utilized in every-day products like cosmetics or hair dyes represent the active substance. In the majority of cases, non-clinical data is already available for these substances, e.g., in the form of technical data sheets. The draft *Guideline on Allergen Product Development for Immunotherapy and Allergy Diagnosis in Moderate to Low-Sized Study Populations* proposes that these already existing data can be compiled for MAA bibliographically. Provided data on acute toxicity, the potential to cause local irritative reactions and the sensitization potential are available, the compilation should usually be sufficient for MAA. If possible, the dataset should be supplemented by bibliographic data on absorption, metabolism, and excretion as well as genotoxicity and carcinogenicity in case of CMR-substances (classified as carcinogenic, mutagenic, or toxic for reproduction). 

While the *EMA Guideline on clinical evaluation of diagnostic agents* [11] can often be applied to products for diagnosis of common type I allergies, this is usually not possible in clinical development of epicutaneous patch test preparations, although, depending on the prevalence of the respective allergy, available patient populations may be of considerable size. However, the guideline cannot be applied in cases where a standard of truth is not available, which in turn is the prerequisite for determination of sensitivity and specificity. In such cases, the current draft of the *Guideline on Allergen Product Development for Immunotherapy and Allergy Diagnosis in Moderate to Low-Sized Study Populations* proposes to analyze alternative parameters such as positive ratio (PR) and reaction index (RI) [8]. The guideline also discusses alternatives to classical dose-finding studies, encompassing epidemiological studies, case reports, published threshold values, data from registers and expert associations and other literature. 

## Future changes in regulatory framework 

As highlighted above, the specific characteristics and challenges associated with allergen products in general and with diagnostic allergen products in particular have been acknowledged by regulators for many years. Continuous efforts are being made to ensure a balance between the necessary regulatory requirements (in particular assurance of quality, safety, efficacy) and the exceptional nature of allergen products. The *Guideline on Allergen Product Development for Immunotherapy and Allergy Diagnosis in Moderate to Low-Sized Study Populations* represents the most recent of these efforts [8]. It has been specifically drafted to provide guidance in cases where adequate data according to existing guidelines cannot be reasonably obtained due to a low number of patients available for clinical trials. This novel EMA/CHMP guideline covers not only the development of therapeutic allergen products, but also specifically addresses products intended for diagnosis of type I and type IV allergies. Its draft will be in public consultation until May 31, 2024 and all stakeholders have been invited to participate in its consultation. Notably, many aspects described in the guideline are already commonly applied by the Paul-Ehrlich-Institut in the national regulation of allergen products in Germany. 

## Disclaimer 

The views expressed in this review are the personal views of the authors and may not be understood or quoted as being made on behalf of or reflecting the position of the respective national competent authority, the European Medicines Agency, or one of its committees or working parties. 

## Authors’ contributions 

Both authors, JZ and VM, conceptualized, wrote and approved the article. 

## Conflict of interest 

The authors declare that they do not have a conflict of interest with regard to this article. 

**Table 1.** Main regulatory documents applicable for allergen products for in vivo diagnosis of type I and type IV allergy.

**Documents**		**Ref.**	**Applicable to test allergens for diagnosis of**	
			**Type I**	**Type IV**
Directive 2001/83/EC		[15]	Yes	Yes
**Quality**				
**European Pharmacopoeia**			Yes	Yes
Monograph on Allergen Products	1063	[19]	Yes	No
Allergen Source Material Monographs	2621, 2623, 2625, 2626, 2627	[20, 21,17], 18, 16	Yes	No
**EMA Guidelines**				
Guideline on Allergen Products: Production and Quality Issues	EMEA/CHMP/BWP/304831/2007	[9]	Yes	No
**ICH Guidelines**	e.g. ICH Q2 (R1), ICH Q5, ICH Q6B		Yes	Yes
**Good Manufacturing Practice**			Yes	Yes
EU Guidelines to Good Manufacturing Practice			Yes	Yes
GMP Directive	Directive 2017/1572	[5]	Yes	Yes
Recommendations on common regulatory approaches for allergen products	CMDh/399/2019	[2]	Yes	Yes
**Clinica**l				
**EMA guidelines**				
Guideline on Clinical Evaluation of Diagnostic Agents	CPMP/EWP/1119/98/Rev 1	[11]	Yes	Yes
Guideline on Clinical Trials In Small Populations	CHMP/EWP/83561/2005	[10]	Yes	Yes
Guideline on Missing Data in Confirmatory Clinical Trials	CPMP/EWP/1776/99 Rev. 1	[12]		
**ICH Guidelines**	e.g., ICH E6 (R1), ICH E9 (R1)		Yes	Yes
**Good Clinical Practice**			Yes	Yes
GCP Directive	2005/28/EC	[6]	Yes	Yes
Clinical Trials Regulation	536/2014	[33]	Yes	Yes
**IN PREPARATION**				
Draft Guideline on Allergen Product Development for Immunotherapy and Allergy Diagnosis in Moderate to Low-Sized Study Populations	EMA/CHMP/72790/2024	[8]	Yes	Yes

BWP = Biologics Working Party; CHMP = Committee for Medicinal Products for Human Use; CMDh = Coordination Group for Mutual Recognition and Decentralized Procedures – Human; EC = European Commission; EMA/EMEA = European Medicines Agency; EWP = Efficacy Working Party; GMP = Good Manufacturing Practice; ICH = International Council for Harmonization of Technical Requirements for Registration of Pharmaceuticals for Human Use.
